# Left Juxtaposition of the Right Atrial Appendage: Pitfalls in Diagnosis

**DOI:** 10.1155/2023/1385305

**Published:** 2023-11-09

**Authors:** Kanya Singhapakdi, Wesam Sourour, Thomas R. Kimball

**Affiliations:** Department of Pediatric Cardiology, Children's Hospital New Orleans, Louisiana State University Health Sciences Center, 200 Henry Clay Ave, New Orleans, Louisiana, USA 70118

## Abstract

Several congenital anomalies of the right atrial appendage (RAA) have been described including aneurysm, herniation (in association with a pericardial defect), and left juxtaposition. The left juxtaposition of the RAA (LJRAA), first described by Birmingham in 1893 and subsequently introduced by Dixon in 1954, is usually associated with complex cardiac malformations such as obstruction of the left ventricular outflow tract. In this case report, we will describe an unusual variant of LJRAA in the absence of any other cardiac defects, which was initially misinterpreted as an aortic dissection. The correct diagnosis was made after careful reinterpretation and the use of multiple imaging modalities as highlighted.

## 1. Introduction

Several congenital anomalies of the right atrial appendage (RAA) have been described including aneurysm [1–3], herniation (in association with a pericardial defect) [4], and left juxtaposition [5]. The left juxtaposition of the RAA (LJRAA), first described by Birmingham in 1893 [6] and subsequently introduced by Dixon in 1954 [7], is usually associated with complex cardiac malformations such as obstruction of the left atrial outlet or aortic outflow tract [8]. In this case report, we will describe an unusual variant of LJRAA in the absence of any other cardiac defects, which was initially misinterpreted as an aortic dissection. The correct diagnosis was made after careful reinterpretation and the use of multiple imaging modalities, as highlighted below.

## 2. Case Report

A five-year-old child with a known history of bicuspid aortic valve (BAV), mild aortic valve stenosis, and mild enlargement of the aortic root presented to their cardiologist at an outside institution for routine follow-up. The patient was otherwise healthy with no known personal or family history of connective tissue disorders. The patient was in their usual state of health with no chest pain, shortness of breath, palpitations, fatigue, or syncope. An electrocardiogram (ECG) performed at the outside institution demonstrated sinus arrhythmia and was otherwise within normal limits and unchanged from prior ECGs.

**Figure 1:**
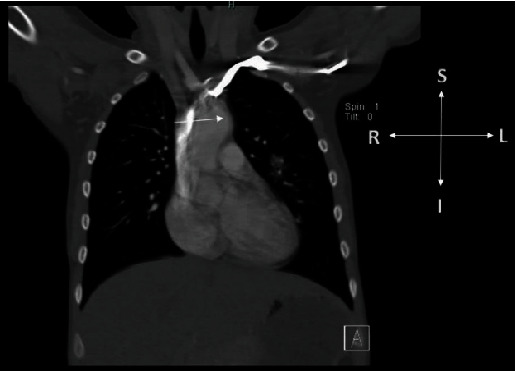
Initial chest CT, coronal view demonstrates what was believed to be a dissection flap (arrow) extending superiorly from the level of the anterior cusp of the aorta.

A transthoracic echocardiogram (TTE) was obtained by the primary cardiologist which demonstrated the known BAV and enlargement of the aortic root and ascending aorta. There were also findings in the suprasternal notch view concerning an intimal flap in the ascending aorta just proximal to the branching of the head and neck vessels. This was suggestive of a possible aortic dissection or aneurysm. Due to these findings, a chest computed tomography (CCT) was obtained. The CT was also concerning for a dissection flap extending from the level of the anterior aortic cusp distally for 2.7 cm (Figure 1). Given these findings, the patient was referred emergently to our institution for further management.

**Figure 2:**
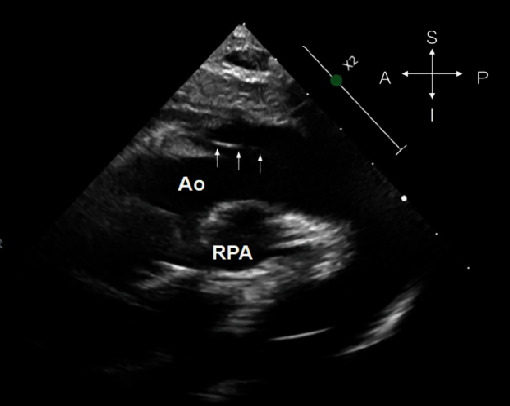
2D TTE, suprasternal view demonstrates a dilated ascending aorta (Ao) and what was thought to be an intimal dissection flap (white arrows). RPA: right pulmonary artery.

**Figure 3:**
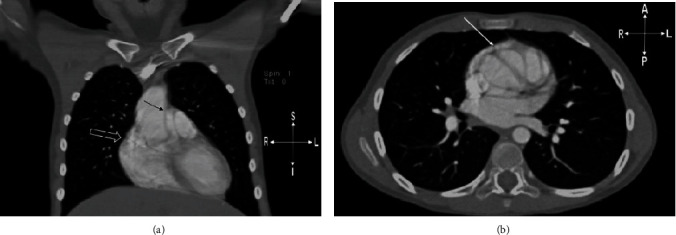
Repeat chest CT, coronal (a) and axial (b) views demonstrate a dilated ascending aorta (21.8 mm; Boston *Z*-score 2.84) and a suspected intimal dissection flap extending anteriorly off of the anterior cusp (thin arrow) just above the sinotubular junction. For comparison, the normal anatomic location for the RAA is highlighted (open arrow).

Upon admission, the patient was in no acute distress and denied any chest pain. Repeat ECG demonstrated normal sinus rhythm with left axis deviation. A repeat TTE (Figure 2) was obtained which again demonstrated what was interpreted as an intimal flap suggestive of an ascending aortic dissection. A repeat CT was interpreted as showing an ascending aortic aneurysm extending anteriorly from the anterior cusp starting just above the sinotubular junction and extending obliquely along the left side of the ascending aorta and just proximal to the transverse arch (Figures 3(a) and 3(b)). The quality of the CT images is suboptimal, as contrast is seen filling both the right and left heart chambers and is not timed to the left side to specifically delineate the left-sided structures of interest. There is also evidence of motion artifact. In retrospect, it is possible the corresponding axial images of this CT may have been reviewed, which would have helped to better delineate the LJRAA vs. an abnormal structure or motion artifact.

**Figure 4:**
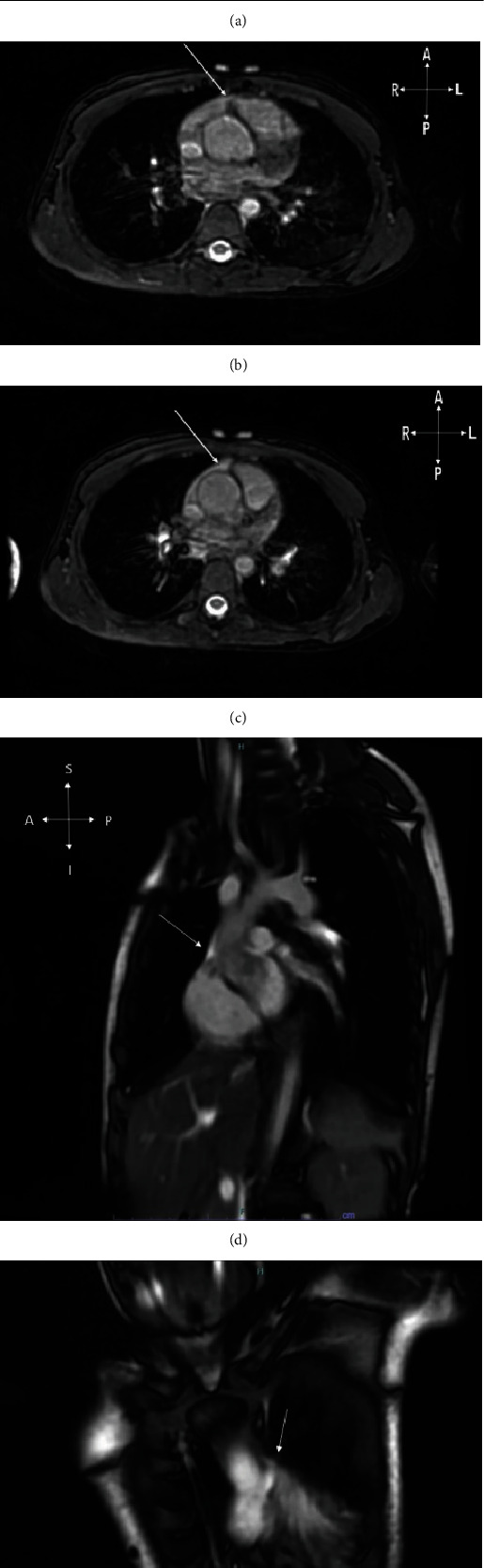
Noncontrast CMR obtained in axial (a–c), sagittal (d), and coronal views (e). Sequential imaging obtained in 3D Heart Navigator Sequence during systole of the aortic root was performed moving from more cephalad to more caudad. The aortic root is intact, and there is no evidence of aneurysm as suspected on CCT. Instead, the arrows in each image highlight the unusual course of the RAA anteriorly around the root of the aorta extending to the left side and account for the suspected intimal flap previously noted.

At this point, the differential was broad and included an aortic dissection, duplication artifact, and left-sided juxtaposed RAA. Juxtaposed atrial appendages can also be confused with ASD if the abnormal septal configuration is not recognized or carefully delineated [5, 9, 10]. Due to the need for a timely and definitive diagnosis, further testing was conducted. Cardiovascular magnetic resonance (CMR) was obtained (Figures 4(a)–4(c)) which demonstrated that what was initially thought to be a dissection was in fact the LJRAA. Interestingly, the right atrial appendage was unusually displaced, coursing leftward and anterior to the aorta. Further reinterpretation of the CT scan confirmed a congenital RAA variation which accounted for the findings described above.

## 3. Discussion

**Figure 5:**
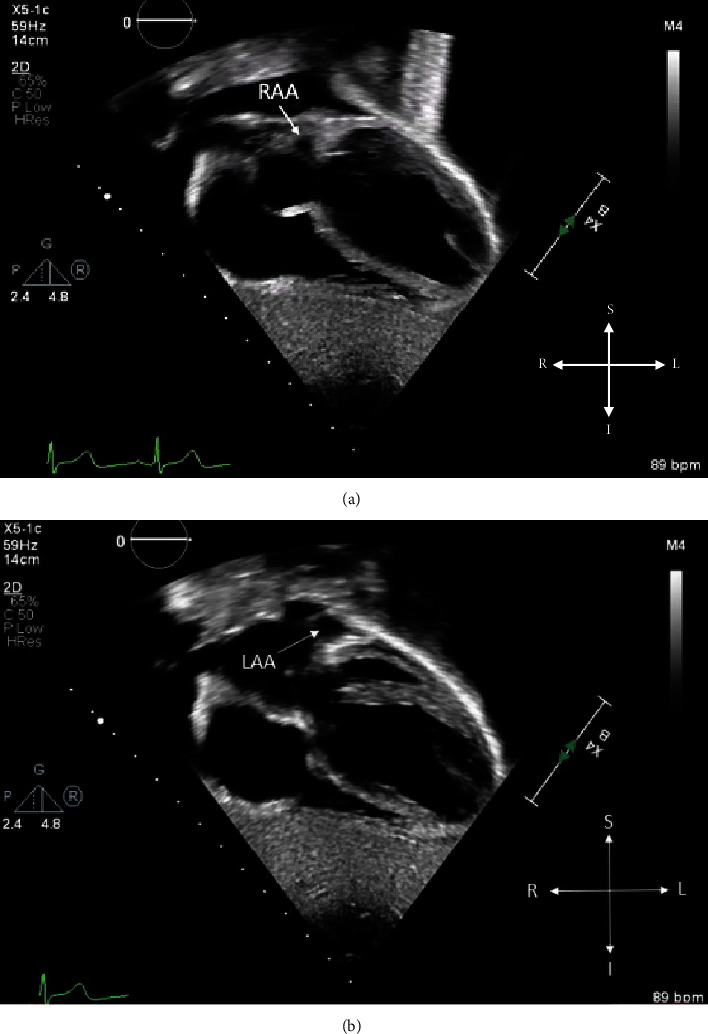
(a, b) 2D TTE, subcostal window, coronal view demonstrates the LJRAA (thin arrow) to the left of the aorta and the normally positioned LAA.

The atrial appendages normally lie on opposite sides of the roots of the great arteries. This patient's anomaly is known as LJRAA, in which both atrial appendages are arranged beside one another and course to the left side of the great arteries (Figure 5). This case represented an unusual variation; in patients with LJRAA, the right atrial appendage usually lies posterior to the aorta as it courses leftward [11]. In our patient, the right atrial appendage was more elongated and laid anterior to the aorta as it coursed leftward.

The left juxtaposition of the RAA is caused embryologically by the underdevelopment of the primitive cardiac tube. In rare instances, one of the appendages may be bifid, with one component in a normal position and the other component juxtaposed. This variant is called partial juxtaposition. CT and CMR in our patient highlighted the entirety of the atrial appendage, thus making a partial juxtaposed appendage less likely.

A review of the literature reveals that juxtaposition of the right atrial appendage in which the appendage courses leftward and anterior to the aorta is rare but has been described; however, it is usually associated with other cardiac defects such as double outlet right ventricle, tricuspid atresia, hypoplastic right ventricle, or an abnormal conus [12, 13], unlike in our case where the LJRAA existed in isolation. Although not hemodynamically significant, the various forms of juxtaposition of the atrial appendages are important to note as they may have implications for catheter-based interventions as well as surgical procedures.

Although TEE is ideal for initial testing and diagnosis, it is highly interpreter-dependent and is associated with multiple ultrasound artifacts. In order to make an accurate and timely diagnosis of rare conditions such as LJRAA, several precautions should be undertaken. Using ECG-gated congenital or structural cardiac-dedicated CT protocols performed and interpreted by congenitally trained imaging experts would be of tremendous value and may have yielded the correct diagnosis earlier. This protocol would have allowed for 3D volume-rendered whole-heart reconstructions with a higher likelihood of greater understanding of the complex congenital anatomy. Alternatively, the use of transesophageal echocardiography (TEE) including 3D with or without ultrasound-enhancing agents may have provided adequate anatomic data. Despite the conventional wisdom that TEE has the benefit of no radiation exposure and therefore a lower risk profile, in young children, this procedure would require sedation, whereas the CCT may not. Finally, the careful stepwise approach taken in this patient was fostered by the discrepancy between the critically reported imaging findings and the asymptomatic clinical state of the patient.

## 4. Conclusion

The misinterpretation of the first echocardiogram described in this case led to an interpretation bias. This highlights how important it is for sonographers, cardiologists, and radiologists to maintain an index of suspicion for varying anatomical configurations of the atrial appendages and their potential to be confused for pathology in order to spare patients from unnecessary radiation, invasive procedures, hospitalization, and further testing. The case also demonstrates the need for the care team to maintain an open mind when going through the diagnostic process and refrain from bias in interpretation based on previously conducted tests.
